# Association between Aerobic Exercise and High-Density Lipoprotein Cholesterol Levels across Various Ranges of Body Mass Index and Waist-Hip Ratio and the Modulating Role of the Hepatic Lipase rs1800588 Variant

**DOI:** 10.3390/genes10060440

**Published:** 2019-06-10

**Authors:** Yasser Nassef, Oswald Ndi Nfor, Kuan-Jung Lee, Ming-Chih Chou, Yung-Po Liaw

**Affiliations:** 1 Institute of Medicine, Chung Shan Medical University, Taichung City 40201, Taiwan; yasser@nassef.org; 2 Department of Public Health and Institute of Public Health, Chung Shan Medical University, Taichung City 40201, Taiwan; nforoswald2@yahoo.com (O.N.N.); jasminemachi@gmail.com (K.-J.L.); 3 Department of Family and Community Medicine, Chung Shan Medical University Hospital, Taichung City 40201, Taiwan

**Keywords:** high-density lipoprotein, aerobic exercise, body fat, hepatic lipase, Taiwan Biobank

## Abstract

Changes in concentrations of high-density lipoprotein cholesterol (HDL-C) are modified by several factors. We examined the relationship between aerobic exercise and HDL-C among different categories of body mass index (BMI) and waist-hip ratio (WHR) and the impact of rs1800588 variant in the hepatic lipase (LIPC) gene. We analyzed data from 6184 men and 8353 women aged 30–70 years. Participants were grouped into two WHR categories: Normal (0 < WHR < 0.9 for men and 0 < WHR < 0.8 for women) and abnormal (WHR ≥ 0.9 for men and WHR ≥ 0.8 for women). The BMI categories were: Underweight (BMI < 18.5 kg/m^2^), normal weight (18.5 ≤ BMI < 24 kg/m^2^), overweight (24 ≤ BMI < 27 kg/m^2^), and obese (BMI ≥ 27 kg/m^2^). Multivariate linear regression models were used to investigate associations between HDL-C and exercise. Aerobic exercise was significantly associated with higher HDL-C (β = 1.18325; *p* < 0.0001) when compared with no exercise. HDL-C was significantly lower in persons with abnormal compared to those with normal WHR (β = −3.06689; *p* < 0.0001). Compared with normal weight, overweight and obese categories were associated with lower HDL-C, with β values of −4.31095 and −6.44230, respectively (*p* < 0.0001). Unlike rs1800588 CT and TT genotypes, associations between aerobic exercise and HDL were not significant among CC carriers no matter their BMI or WHR.

## 1. Introduction

Prospective cohort studies have consistently demonstrated that high-density lipoprotein-cholesterol (HDL-C) is a strong predictor of cardiovascular diseases in different populations [1,2,3]. It has antioxidative, anti-inflammatory, antidiabetic, and anti-thrombotic activities [4,5], and plays an essential role in the management of coronary heart disease (CHD) and risk reduction [6]. A higher level of HDL-C is protective against heart disease. On the other hand, lower levels HDL-C (defined as <40 mg/dL in men and <50 mg/dL in women) [7] are associated with higher risks for heart disease.

Environmental and genetic factors contribute to variations in HDL-C levels. Exercise training is one of the strategies suggested to improve HDL-C function via proprotein convertase subtilisin/kexin type 9 (PCSK9) and/or sterol regulatory element binding protein 2 (SREBP2) [1]. Besides modifying HDL subclass distribution, exercise training has also resulted in a decrease in the body mass index of obese women [4]. Aerobic exercise has been recommended for the prevention of coronary heart disease [1,8], which is a serious health issue in Taiwan. This exercise training also reduces stress, has fewer side effects compared to medications, and is easier to carry out [1]. Of note, many studies have investigated the benefits of exercise on HDL-C. In our recently published study, we found that aerobic exercise was associated with a higher level of HDL-C (β = 1.3154; *p* < 0.0001) among Taiwanese adults [9]. Findings from a previous study have suggested that compared to other lipid fractions, HDL-C levels are more sensitive to aerobic exercise [1]. 

Several variants have been associated with HDL-C [10]. The hepatic lipase (LIPC) gene located on chromosome 15 (q21–q23) influences the production of the hepatic lipase enzyme that plays a vital role in lipid metabolism [11]. Based on previous data, genetic variation in hepatic lipase activity is an important determinant of plasma HDL-C concentrations [12]. Rs1800588, a common variant in the LIPC gene has been associated with a higher concentration of HDL-C [13]. Prior work involving female carriers of this variant demonstrated that those carrying at least one copy of the minor allele had higher HDL- levels than those that were homozygous for the major allele [14]. In addition, variations in several genes (including the LIPC gene) are reported to influence interindividual variability in the HDL-C response to exercise [14]. However, the impact of physical exercise on the relationship between hepatic lipase activity and HDL-C levels has not been reported in Taiwan. 

Obesity indices including body mass index (BMI) and waist-hip ratio (WHR) increase with increasing categories of abnormal serum lipids [15]. BMI is defined as a person’s weight in kilograms divided by the square of height in meters while WHR is a dimensionless ratio that is calculated as the waist circumference divided by the hip circumference. Among the anthropometric measures, WHR has shown good correlations with serum lipids especially among elderly women [16]. On the other hand, negative associations have been found between BMI and HDL-C [17]. Based on previous literature, significant differences have been found between pre and post-test values of HDL-C, BMI, and WHR of individuals who were engaged in 8 weeks of aerobic training [18]. Considering that BMI and WHR are independently associated with HDL-C and that aerobic training has improved both anthropometric variables, we investigated the association between aerobic exercise and HDL-C across different categories of BMI and WHR. Furthermore, we tested whether this association is modified by a selected HDL-C raising variant (LIPC rs1800588). 

## 2. Materials and Methods

### 2.1. Data Source

Phenotypic and genotypic data were collected from participants (aged 30–70 years) that were enrolled in Taiwan Biobank from 2008–2016. Recruitment methods in the Biobank are in accordance with relevant guidelines and regulations. Written informed consents are obtained from all participants prior to data collection. Data collection was through questionnaires as well as physical and biochemical examinations. The Institutional Review Board of Chung Shan Medical University approved this study (project identification code CS2-16114).

### 2.2. Study Participants

We analyzed data from 6184 men and 8353 women aged 30–70 years recruited in the Taiwan Biobank project from 2012–2016. Age, sex, BMI, WHR, and lifestyle (physical activity, coffee drinking, smoking, alcohol consumption, and vegetarian diet) measures were determined from the database. Participants were grouped into WHR categories as follows: Normal (0 < WHR < 0.9 for men and 0 < WHR < 0.8 for women), and abnormal (WHR ≥ 0.9 for men and WHR ≥ 0.8 for women). Likewise, the BMI categories included the following: Normal weight (18.5 ≤ BMI < 24 kg/m^2^), overweight (24 ≤ BMI < 27 kg/m^2^), and obesity (BMI ≥ 27 kg/m^2^). Information on aerobic exercise was self-reported. Using questionnaires in the Biobank, participants selected at most 3 types of their habitual aerobic activities, which included jogging, strolling, swimming, yoga, taijiquan, biking, and aerobic dance. The minimum amount of exercise was 30 min per session, at least 3 times per week, for the last 3 months. “No exercise” was defined as participation in exercise for less than 30 min per day and less than two times per week.

### 2.3. SNP Selection and Genotyping

We selected the LIPC variant (rs1800588) that has been consistently associated with elevated levels of HDL-C through a literature search. Genotyping was performed using TaqMan SNP Genotyping Assays from Applied Biosystems (ABI; Foster City, CA, USA). We included only participants with call rates greater than 90%. Polymorphic variants with minor allele frequency (MAF) <0.05, as well as those whose genotypes deviated from the Hardy-Weinberg equilibrium (HWE) were excluded.

### 2.4. Statistical Analysis

Analyses were performed using the SAS 9.4 software (SAS Institute, Cary, NC, USA). Differences in HDL-C among the body fat indicators (BMI and WHR) were compared using the *t*-test. The association between HDL-C and exercise was determined using multivariate linear regression models. Data were presented as mean ± standard error (SE) for continuous variables. Values of *p* < 0.05 were considered statistically significant.

## 3. Results

Average levels of HDL-C in study participants were determined among different categories of WHR and BMI as shown in Table 1 and Table 2. Among participants who were engaged in aerobic exercise, HDL level was 56.43 ± 0.35 mg/dL in those with normal WHR and 53.89 ± 0.23 mg/dL in those with abnormal WHR (*p* < 0.0001). Mean HDL levels differed significantly among the different categories of BMI (*p* < 0.0001). Individuals who had aerobic exercise had higher HDL-C than those who did not exercise. That is 66.83 ± 1.55 mg/dL vs. 65.32 ± 0.80 mg/dL for underweight; 58.60 ± 0.28 mg/dL vs. 57.61 ± 0.19 mg/dL for normal weight; 52.02 ± 0.32 mg/dL vs. 49.51 ± 0.21 mg/dL for overweight; and 47.30 ± 0.35 mg/dL vs. 46.49 ± 0.23 mg/dL for the obese category. The overall effect of aerobic exercise on HDL-C is shown in Table 3. Aerobic exercise was significantly associated with higher HDL-C (β = 1.18325; *p* < 0.0001) when compared with no exercise. HDL-C was significantly lower in persons with abnormal compared to those with normal WHR (β = −3.06689; *p* < 0.0001). Compared with normal weight individuals, overweight and obese groups were also associated with lower HDL-C, with β values of −4.31095 and −6.44230, respectively (*p* < 0.0001). Table 4 is the association of HDL-C based on WHR. There was an interaction between WHR and aerobic exercise on HDL-C (*p* = 0.0421). After the stratification, aerobic exercise was associated with a higher HDL-C especially in those with normal WHR (β = 1.69668, *p* < 0.0001 vs. 0.97921, *p* < 0.0001). Table 5 is an association of HDL-C with aerobic exercise based on BMI. After stratification by BMI, significant associations of aerobic exercise and HDL-C were found only for normal (β = 1.03261, *p* = 0.0019) and overweight (β = 2.01758, *p* < 0.0001) categories. Rs1800588 CT and TT carriers who had aerobic exercise were associated with a higher HDL-C compared to their inactive counterparts. That is, significant increases in HDL were noticed only among aerobically active CT carriers with normal weight (β = 1.99961, *p* = 0.0027), overweight (β = 1.59362, *p* = 1.1371), and abnormal WHR (β = 1.48073, *p* = 0.0063), as well as in TT carriers with both normal and abnormal WHR (β = 4.04073, *p* = 0.0094 and β = 2.19244, *p* = 0.0445), and those in the overweight category (5.54693, *p* = 0.0003) (Table 6). 

## 4. Discussion

The primary objective of this study was to determine the association between aerobic exercise and HDL-C among different categories of BMI and WHR and also to highlight the modulating role of rs1800588 variant in the hepatic lipase gene. We found that (1) consistent with our previous findings [9], aerobic exercise was better than no exercise for improving HDL-C in Taiwanese adults. (2) Aerobic exercise was associated with a lower HDL-C in persons with abnormal compared to normal WHR (β = −306,689, *p* < 0.0001). In addition, there was an interaction between WHR and aerobic exercise. (3) Compared with aerobically active normal weight individuals, their overweight and obese counterparts were associated with lower HDL-C levels. (4) Unlike the LIPC rs1800588 TT and CT genotype, the effect of CC genotype on HDL was not modified by aerobic exercise no matter the BMI or WHR category. Our study findings highlight the impact of aerobic exercise on HDL-C. This in part is mediated by liver X receptor (LXR) [1] and liver ATP-binding cassette transporters A-1 (ABCA1) [19] as previously reported. 

Previously published articles have discussed associations of HDL-C with anthropometric measures [20] and physical exercise [1,9]. Findings from a study of 28,000 men and women suggested that HDL-cholesterol decreased concurrently with increases in BMI [21]. In another study, WHR was found to be a good predictor of the lipid profile (β = 3.51, *p* = 0.005) [22]. Aerobic exercise has resulted in significant changes in body fat measures like BMI and WHR among young Taiwanese adults who were obese [23]. Despite the numerous findings, the impact of aerobic exercise on HDL levels based on anthropometric measures and genetic factors have not been reported in Taiwan. In the current study, we included rs1800588 variant in the LIPC gene in the model and found that the effect of CC genotype on HDL was not modified by aerobic exercise no matter the BMI or WHR category. However, the magnitude of the association between CT and TT genotypes on HDL-C differed with respect to BMI and WHR categories. For instance, we found that the effect of CT genotype on HDL was significant only among aerobically active normal weight and overweight adults as well as those with abnormal WHR, while the effect of TT genotype on HDL-C was significant only among aerobically active overweight adults and those with both normal and abnormal WHR. The mechanisms explaining these differences in HDL-C response with respect to body fat measures are still to be clearly understood. However, it has been reported that HDL levels of certain individuals do not necessarily increase no matter the exercise regimen [14,24]. Another study including Caucasian women found that the effect of rs1800588 variant on HDL-C was modified by physical activity [14]. However, stratifications were not made based on genotypes. 

The T allele of rs1800588 has been associated with higher baseline levels of HDL-C [25]. As stated earlier female carriers of the rs1800588 variant with at least one copy of the minor allele had higher concentrations of HDL-C than those that were homozygous for the major allele [14]. Further analysis of data from those women demonstrated that the per-minor allele increase in HDL-C was greater in active than inactive women. This aligns with other findings which suggested that LIPC polymorphisms might serve as useful indicators of higher HDL-C in women [25,26]. Prior findings from studies investigating the relationship between LIPC rs1800588 and HDL-C differ according to gender and ethnicity [11]. In our study, there was the presence of LIPC rs1800588 CC, CT and TT genotypes. However, in a study by Brinkley and his colleagues, there were no subjects with the rs1800588 TT genotype [25]. This highlights the diverse effect of the variant on HDL-C levels.

Anthropometric, lifestyle, environmental, and genetic factors influence changes in Lipid fractions [1]. Moderate intensity aerobic exercise is related to a higher HDL-C [9]. Variations in HDL-C responses to exercise are influenced by several factors including sex, changes in body composition and genetic effects [27]. Based on our analyzed data, there was a significant LIPC rs1800588*exercise effect on HDL-C particularly in overweight adults, with higher levels in CT and TT compared to CC carriers (*p* = 0.0281 for the interaction). There were no genotype*exercise interactions for HDL-C across other BMI and WHR categories. 

In summary, we report evidence that associations between aerobic exercise and HDL-C levels in Taiwanese adults differed not only across different ranges of body mass index and waist-to-hip ratios but also among carriers of the rs1800588 variant located in the hepatic lipase gene. However, unlike CT and TT genotypes, the effects of aerobic exercise on HDL-C levels were not significant among rs1800588 CC carriers no matter their BMI or WHR.

## Figures and Tables

**Table 1:** Mean HDL-C levels of participants categorized by waist-hip ratio (WHR).

	Normal WHR		Abnormal WHR		*p*-Value
	(n = 5190)		(n = 9347)		
	n	Mean ± SE	n	Mean ± SE	
Exercise					
No exercise	3565	55.01 ± 0.23	6148	52.13 ± 0.16	<0.0001
Aerobic	1625	56.43 ± 0.35	3199	53.89 ± 0.23	<0.0001
BMI					
Underweight	276	66.49 ± 0.87	132	63.98 ± 1.23	0.0993
Normal	3159	58.28 ± 0.24	3799	57.67 ± 0.21	0.0587
Overweight	1332	49.38 ± 0.30	2939	50.84 ± 0.22	<0.0001
Obese	423	46.32 ± 0.48	2477	46.80 ± 0.21	0.3770
Body fat rate					
Normal	3941	56.50 ± 0.22	3600	55.00 ± 0.23	<0.0001
Abnormal	1249	52.15 ± 0.36	5747	51.31 ± 0.16	0.0330
Sex					
Women	2096	62.74 ± 0.30	6257	56.46 ± 0.16	<0.0001
Men	3094	50.52 ± 0.21	3090	45.19 ± 0.18	<0.0001
Age, year					
30–40	2066	55.56 ± 0.30	2046	52.06 ± 0.28	<0.0001
40–50	1495	55.26 ± 0.36	2592	52.86 ± 0.26	<0.0001
51–60	1141	55.70 ± 0.42	3000	53.11 ± 0.24	<0.0001
61–70	488	55.07 ± 0.63	1709	52.68 ± 0.31	0.0004
Smoking					
Never	3980	57.06 ± 0.22	7489	54.29 ± 0.15	<0.0001
Former	603	51.30 ± 0.48	946	47.50 ± 0.37	<0.0001
Current	307	49.09 ± 0.50	912	45.37 ± 0.37	<0.0001
Drinking					
Never	4720	55.72 ± 0.20	8464	53.15 ± 0.14	<0.0001
Former	107	49.41 ± 1.05	261	44.30 ± 0.65	<0.0001
Current	363	53.85 ± 0.67	622	50.64 ± 0.54	0.0003
Coffee drinking					
No	3380	55.01 ± 0.23	6322	52.03 ± 0.16	<0.0001
Yes	1810	56.29 ± 0.33	3025	54.20 ± 0.24	<0.0001
Vegetarian diet					
Non	4729	55.69 ± 0.20	8418	52.93 ± 0.14	<0.0001
Former	227	55.08 ± 0.96	458	52.53 ± 0.63	0.0234
Current	234	51.01 ± 0.74	471	49.38 ± 0.56	0.0841

SE = standard error, BMI = body mass index, HDL-C = high-density lipoprotein cholesterol.

**Table 2:** Mean HDL-C levels of participants categorized by BMI.

	Underweight		Normal Weight		Overweight		Obese		*p*-Value
	(n = 408)		(n = 6958)		(n = 4271)		(n = 2900)		
	n	Mean ± SE	n	Mean ± SE	n	Mean ± SE	n	Mean ± SE	
Exercise									
No exercise	312	65.32 ± 0.80	4563	57.61 ± 0.19	2791	49.51 ± 0.21	2047	46.49 ± 0.23	<0.0001
Aerobic	96	66.83 ± 1.55	2395	58.60 ± 0.28	1480	52.02 ± 0.32	853	47.30 ± 0.35	<0.0001
Waist-hip ratio									
Normal	276	66.49 ± 0.87	3159	58.28 ± 0.24	1332	49.38 ± 0.30	423	46.32 ± 0.48	<0.0001
Abnormal	132	63.98 ± 1.23	3799	57.67 ± 0.21	2939	50.84 ± 0.22	2477	46.80 ± 0.21	<0.0001
Body fat rate									
Normal	408	65.68 ± 0.71	5191	58.20 ± 0.19	1686	47.64 ± 0.26	256	44.66 ± 0.63	<0.0001
Abnormal	0	-	1767	57.20 ± 0.30	2585	52.17 ± 0.23	2644	46.93 ± 0.20	<0.0001
Sex									
Women	333	66.55 ± 0.79	4766	60.72 ± 0.19	1963	54.66 ± 0.27	1291	51.06 ± 0.30	<0.0001
Men	75	61.83 ± 1.52	2192	51.92 ± 0.25	2308	46.75 ± 0.21	1609	43.25 ± 0.21	<0.0001
Age									
30–40	183	64.13 ± 1.01	2156	57.83 ± 0.27	941	49.69 ± 0.36	832	45.82 ± 0.35	<0.0001
40–50	99	66.72 ± 1.44	1966	58.60 ± 0.30	1209	49.75 ± 0.33	813	46.35 ± 0.35	<0.0001
51–60	81	67.20 ± 1.66	1846	58.16 ± 0.32	1355	51.19 ± 0.31	859	47.40 ± 0.37	<0.0001
61–70	45	66.96 ± 2.40	990	56.55 ± 0.43	766	50.79 ± 0.45	396	47.96 ± 0.51	<0.0001
Smoking									
Never	375	66.05 ± 0.74	5900	58.97 ± 0.17	3169	51.54 ± 0.21	2025	48.20 ± 0.23	<0.0001
Former	12	59.00 ± 4.60	489	53.24 ± 0.56	604	48.66 ± 0.45	444	44.45 ± 0.44	<0.0001
Current	21	62.86 ± 2.97	569	51.38 ± 0.53	498	45.10 ± 0.48	431	42.13 ± 0.42	<0.0001
Drinking									
Never	392	65.64 ± 0.73	6512	58.10 ± 0.16	3778	50.56 ± 0.19	2502	47.04 ± 0.21	<0.0001
Former	1	62.00	100	51.37 ± 1.16	127	45.49 ± 0.91	140	41.94 ± 0.75	<0.0001
Current	15	67.07 ± 3.73	346	56.97 ± 0.76	366	50.26 ± 0.66	258	46.26 ± 0.60	<0.0001
Coffee drinking									
No	301	65.30 ± 0.79	4648	57.21 ± 0.19	2834	49.60 ± 0.21	1919	46.24 ± 0.23	<0.0001
Yes	107	66.74 ± 1.56	2310	59.45 ± 0.28	1437	51.93 ± 0.33	981	47.67 ± 0.34	<0.0001
Vegetarian diet									
No	364	66.12 ± 0.74	6240	58.28 ± 0.17	3900	50.57 ± 0.19	2643	46.91 ± 0.20	<0.0001
Former	9	69.22 ± 5.18	343	58.69 ± 0.76	183	49.02 ± 0.79	150	45.59 ± 0.85	<0.0001
Current	35	60.20 ± 2.54	375	51.76 ± 0.60	188	47.82 ± 0.75	107	43.79 ± 0.97	<0.0001

**Table 3:** Overall effect of aerobic exercise, WHR, and BMI on HDL-C levels.

	β-Coefficient	*p*-Value
Exercise (ref: No exercise)		
Aerobic	1.18325	<0.0001
Waist-hip ratio (ref: Normal)		
Abnormal	−3.06689	<0.0001
BMI (ref: Normal)		
Underweight	5.64809	<0.0001
Overweight	−4.31095	<0.0001
Obese	−6.4423	<0.0001
Body fat rate (ref: Normal)		
Abnormal	−2.1956	<0.0001
Sex (ref: Women)		
Men	−9.87778	<0.0001
Age (ref: 30–40)		
40–50	0.75841	0.003
51–60	1.18484	<0.0001
61–70	0.87361	0.0068
Smoking (ref: Never)		
Former	−0.16071	0.6368
Current	−2.92965	<0.0001
Drinking (ref: Never)		
Former	−1.35588	0.0292
Current	4.0583	<0.0001
Coffee drinking (ref: No)		
Yes	1.29158	<0.0001
Vegetarian diet (ref: No)		
Former	−0.88308	0.0484
Current	−5.69481	<0.0001

**Table 4:** Multiple linear regression showing the effect of aerobic exercise on HDL-C based on WHR.

	Normal WHR		Abnormal WHR	
	β	*p*-Value	β	*p*-Value
Exercise (ref: No exercise)				
Aerobic	1.69668	<0.0001	0.97921	0.0002
BMI (ref: Normal)				
Underweight	5.59243	<0.0001	5.02426	<0.0001
Overweight	−5.27181	<0.0001	−3.73275	<0.0001
Obese	−7.09311	<0.0001	−6.20423	<0.0001
Body fat rate (ref: Normal)				
Abnormal	−2.42606	<0.0001	−2.10034	<0.0001
Sex (ref: Women)				
Men	−10.28598	<0.0001	−9.68871	<0.0001
Age (ref: 30–40)				
40–50	0.63513	0.1145	0.8325	0.0128
51–60	1.43776	0.0015	1.04859	0.0017
61–70	1.37112	0.0267	0.71197	0.0659
Smoking (ref: Never)				
Former	−0.61703	0.2593	0.10417	0.8122
Current	−3.36699	<0.0001	−2.6385	<0.0001
Drinking (ref: Never)				
Former	−2.05145	0.0784	−1.29173	0.0784
Current	3.60076	<0.0001	4.30474	<0.0001
Coffee drinking (ref: No)				
Yes	0.80019	0.0203	1.59424	<0.0001
Vegetarian diet (ref: No)				
Former	−1.0724	0.178	−0.79071	0.1425
Current	−6.41356	<0.0001	−5.30493	<0.0001
WHR*exercise			*p*-value = 0.0421	

β = β value, ref. = reference.

**Table 5:** Multiple linear regression showing the effect of aerobic exercise on HDL-C based on BMI.

	Underweight		Normal Weight		Overweight		Obese	
	β	*p*-Value	β	*p*-Value	β	*p*-Value	β	*p*-Value
Exercise (ref: No exercise)								
Aerobic	0.25906	0.8905	1.03261	0.0019	2.01758	<0.0001	0.45652	0.2547
WHR (ref: Normal)								
Abnormal	−4.15959	0.0097	−3.93381	<0.0001	−2.04105	<0.0001	−1.81686	0.0004
Body fat rate (ref: Normal)								
Abnormal	-	-	−3.48363	<0.0001	−0.44724	0.3138	−1.17241	0.0718
Sex (ref: Women)								
Men	−6.10877	0.0038	−10.93384	<0.0001	−9.12807	<0.0001	−7.89031	<0.0001
Age (ref: 30–40)								
40–50	4.01615	0.0298	1.37246	0.0004	−0.10543	0.8231	0.32885	0.4854
51–60	4.58714	0.0229	1.63715	<0.0001	0.60932	0.2041	0.90307	0.0615
61–70	4.24658	0.1094	1.03037	0.0436	0.65788	0.2371	1.17587	0.0531
Smoking (ref: Never)								
Former	−6.12763	0.1880	−1.08682	0.0830	0.75294	0.1476	−0.1813	0.7427
Current	−2.77427	0.4150	−3.40832	<0.0001	−2.86523	<0.0001	−2.51485	<0.0001
Drinking (ref: Never)								
Former	−2.20863	0.8829	−1.67239	0.1854	−1.57299	0.1130	−1.55493	0.0702
Current	5.43379	0.1797	4.53503	<0.0001	4.27903	<0.0001	2.86789	<0.0001
Coffee drinking (ref: No)								
Yes	0.77143	0.6411	1.33778	<0.0001	1.73784	<0.0001	0.74399	0.0472
Vegetarian diet (ref: No)								
Former	2.80844	0.5567	0.11192	0.8694	−2.72699	0.0008	−1.35844	0.0877
Current	−5.40391	0.0338	−7.19076	<0.0001	−3.77391	<0.0001	−4.27177	<0.0001

**Table 6:** The impact of aerobic exercise on HDL-C stratified by rs1800588 variant and obesity indexes.

rs1800588	WHR Stratification				BMI Stratification							
	Normal WHR		Abnormal WHR		Underweight		Normal		Overweight		Obese	
	β	*p*-Value	β	*p*-Value	Β	*p*-Value	β	*p*-Value	β	*p*-Value	β	*p*-Value
CC	1.36476	0.0798	0.78743	0.1622	2.38864	0.5136	0.63897	0.3730	1.22861	0.1093	1.47101	0.1052
CT	1.22236	0.0870	1.48073	0.0063	−1.45958	0.7809	1.99961	0.0027	1.59362	0.0371	0.00127	0.9987
TT	4.04073	0.0094	2.19244	0.0445	15.7138	0.2848	1.18290	0.3920	5.54693	0.0003	2.02256	0.2826
*P*-interaction	0.5865		0.0792		0.4733		0.9596		0.0281		0.9294	

The *p*-interaction shown is for hepatic lipase (LIPC) Rs1800588 and exercise (Rs1800588*exercise).
